# Hodgen’s Dental Metallurgy

**Published:** 1911-11-15

**Authors:** 


HODGEN'S DENTAL METALLURGY. The fourth edition of this excellent work on dental metallurgy has just been issued by the C. V. Mosby Co., of St. Louis. This edition has been completely revised by Prof. Guy S. Millberry, of California University Dental School. It is a well made book of 375 pages and covers the field of dental metallurgy probably as well as any work of its kind. The book has always been a favorite with teachers because it is possible to use it as a class text even in schools which have no special laboratory facilities, but it is especially useful when studied in connection with a laboratory where the text can be supplemented by practical experiments that are easily within the compass of the dental student and the usual college laboratory. Metallurgy as a distinct study of the dental curriculum is not as generally given as it should be. With such a book as this every dental college could readily install a practical course of much value. Prof. Millberry's work on the book shows to advantage largely in the bringing of the text up to date and into harmony with the more recent investigations of scientific chemistry as well as the more popular applied dental chemistry.

We note that in the treatment of gold the use of gold and the methods of purifying it for cast inlays has not been specially considered. This is one of the most important uses of gold today and very few dentists are sufficiently familiar with the manipulations of gold in this process to secure the most desirable results. Some good work along this line has been done by Price and others, which should be noticed in a work of this character.

